# Variation of Phenolics (Bound and Free), Minerals, and Antioxidant Activity of Twenty-Eight Wild Edible Fruits of Twenty-Three Species from Far North Region of Cameroon

**DOI:** 10.1155/2021/4154381

**Published:** 2021-07-12

**Authors:** B. B. Koubala, J. P. Bayang, H. Wangso, M. C. Kolla, A. Laya

**Affiliations:** ^1^Department of Chemistry, Faculty of Science, University of Maroua, P.O. Box 814 Maroua, Cameroon; ^2^Department of Life and Earth Sciences, Higher Teacher's Training College of Maroua, University of Maroua, P.O. Box 55 Maroua, Cameroon; ^3^Department of Biological Sciences, Faculty of Science, University of Maroua, P.O. Box 814 Maroua, Cameroon

## Abstract

The present study is aimed at investigating the variation of phenolics (bound and free), minerals, and antioxidant potentials of the wild edible fruits (fresh and dry) native from Far North Region of Cameroon. The results showed significant (*p* < 0.01) differences among fruits and species for all parameters. Bound phenolic content (mgGAE/100 g) of dry fruits (DF) ranged from 95.58 to 407.72; however, the contents were varied from 28.97 to 306.04 in fresh fruits (FF). Free phenolic content varied from 46.43 to 344.73 in DF and fold from 119.54 to 315.79 for those FF. Flavonoids (4.27-256.87 mg QE/100 g), tannins (3.24-63.42 mg CE/100 g), and anthocyanin content (8.65-168.10 mg C3GE/100 g) in fruits varied also significantly in respect with DF and FF. The mineral content analysis indicates that the wild fruits are rich in valuable macro- and trace elements. For antioxidant activities, except high 2.2-diphenyl-1-picyhydrazyl (DPPH) scavenging activity obtained with free phenolics, the bound phenolics of FF and DF had significantly high ferric reducing antioxidant power (FRAP) and 2,2-azino-bis(3-ethylbenzylthiozoline-6-sulphonic acid) (ABTS) scavenging activity. Furthermore, free and bound phenolic content was highly and positively correlated with ABTS, DPPH, and FRAP activities confirmed by the principal component analysis (F1×F2: 60.17%). The present study revealed that the wild edible fruits of twenty-three species investigated are important sources of bioactive compounds, natural antioxidants, and nutraceutical potential to prevent/to treat chronic diseases which could be benefits for the consumers.

## 1. Introduction

Many countries in the world are paying a lot of price to the well-being of their populations because of chronic noncommunicable disease threats. Although, the preventive strategies have led to some positive results; however, overweight and obesity continue to increase in all regions of the world affecting children and adults. For example, 40 million children under five in the world were overweight, and 44% of overweight children aged 5-9 were obese [1]. In sub-Saharan Africa, 48% had hypertension, 5.1% were diabetics, and 20% were suffering from obesity [2]. In Cameroon, noncommunicable diseases were accounted for 43% of all deaths [3]. Hence, it is urgent to know how traditional plant foods can be used to manage these diseases. Therefore, to better manage this burden, human beings have forgotten that the environment in which they are living provides functional food and medicines such as nonconventional edible fruits. Fact, numerous researches demonstrated that nonconventional edible fruits are good sources of important components with potential biological activities promoting health benefits especially polyphenolic compounds such as flavonoids, anthocyanins, and tannins [4, 5] as well as minerals. For Manach et al. [6], the amounts consumed of the compounds and their bioavailability on their target organs or tissues confer their high antioxidant activities. Thus, these compounds can reduce the risk of inflammatory and degenerative/noncommunicable diseases such as cancer, cardiovascular diseases, stroke, diabetes, hypertension, and obesity [7, 8].

To date, conventional fruits have been largely used instead of the wild edible fruits as the main plant foods; though, the wild edible fruits have been recognized as good nutritional and bioactive nutrients for healthier life of human [9]. Phenolics are found in most fruits and vegetables with a significant portion of the diet [10]. The health benefits of wild edible fruits are attributed to their various phenolic compounds; however, previous works have been conducted only on their soluble fractions [6]. For example, antioxidant and DNA damage protection potentials of phenolic acids have been reported by Sevgi et al. [10], for free phenolics. However, phenolic compounds are found in different forms in plant species (free, bound, or esterified), and their antioxidant efficient depends mostly on these different forms [11–13]. Also, recent studies have shown that phenolic contents in fruits differ greatly according to the ripening stages and harvesting seasons or geographical location [14, 15]. In the case of the wild edible fruits harvested throughout different seasons, there is no information on bound and free phenolic content as well their antioxidant capacities. Furthermore, the nonconventional edible fruits are eating in fresh or dry forms, while others are eating both in their fresh and dry forms. Since these edible forms of nonconventional fruits exist, data reflecting the variation of their bioactive compounds and antioxidant potentials between these edible forms are also very scare in the literature. The wild edible fruits may be important source of bound phenolics, essential minerals, and antioxidant properties as well as nutraceutical potential to reduce diseases.

Therefore, the present study is aimed at investigating the phenolics (free and bound) and minerals of twenty-eight wild edible fruits of twenty-three species harvested both for dry and fresh forms throughout dry and rainy seasons and also to evaluate their antioxidant potentials. To the best of our knowledge, the present study is the first report regarding the contents of bioactive compounds (bound and free) and minerals of the most consumed wild fruits among 68 species collected and linked to the traditional diet of the population from Far North Region of Cameroon.

## 2. Material and Methods

### 2.1. Samples and Sampling Sites

**Figure 1:**
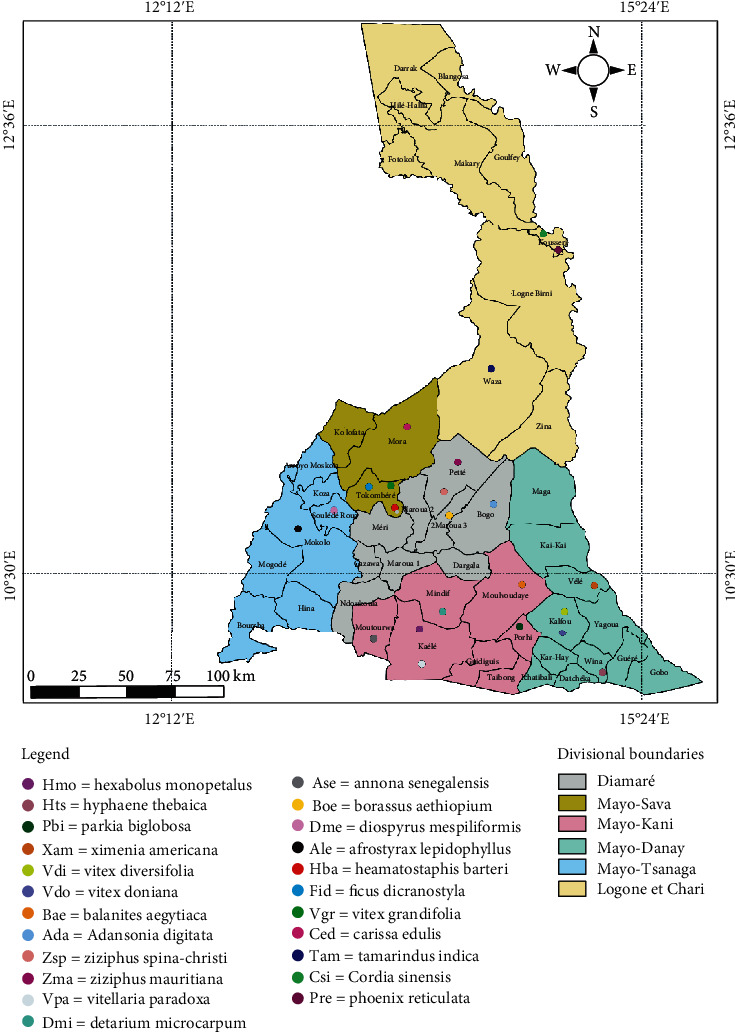
Site location map from Far North Region of Cameroon.

**Figure 2:**
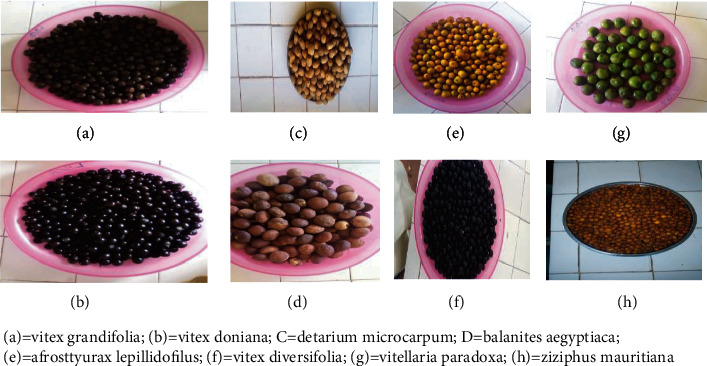
Some samples of wild edible fruits (dry and fresh forms) among 23 species.

Twenty-three species among 68 of wild edible fruits investigated because these are mostly appreciated and consumed, and there are also usually transformed in some byproducts as well for selling across other regions or countries by local population from Far North Region of Cameroon (Figure 1). Fruits were picked throughout the different locations from 23^rd^April 2019 to 25^th^ April 2020 during the dry (November to April: 23-45°C) and rainy (May to October: 20-35°C) seasons. These fruits have been selected because of their many therapeutic effects to treat headache, diabetes types 2, obesity, kidney failure, cancer, and hypertension and have potential antioxidant and antimicrobial properties as results of the survey conducted by our teams in 2019 (Table 1). Two types of fruits forms were used for the present study. Fruits eaten only in the fresh form were *Vitex diversifolia*, *Vitellaria paradoxa*, *Haematostaphis barteri*, *Annona senegalensis*, *Ximenia americana*, *Ziziphus mauritiana, Cordia sinensis*, *Hyphaene thebaica*, *Carissa edulis*, *Ziziphus sipna-christi*, *Ficus dicranostyla*, *Afrostyrax lepidophyllus*, *Hexabolus monopetalus*, *Borassus aethiopium, Diospyrus mespiliformis*, and *Phoenix reclinatum*; however, those eaten in the dry form were *Detarium microcarpum*, *Parkia biglobosa*, *Ziziphus mauritiana*, *Balanites aegyptiaca*, *Diospyrus mespiliformis*, *Tamarindus indica*, *Adansonia digitata*, *Ziziphus spina-christi*, *Phoenix reclinatum*, and *Hyphaene thebaica,* while,fruits from *Z. mauritiana, H. thebaica, Z. sipna-christi, D. mespiliformis, and P. reclinatum* species were consumed both in dry and fresh forms. Eleven species of fruits such as *V. diversifolia, V. paradoxa*, *H. monopetalus*, *B. ethiopium, V. doniana*, *V. grandifolia*, *H. barteri*, *A. senegalensis*, *X. americana*, *A. lepidophyllus*, *and C. edulis* were harvested in rainy season, whereas twelve other fruits were harvested in the dry season. Fresh fruits were harvested at the last stage of the maturity; however, the dry fruits were picked in dried form from the trees plant directly. Some samples of twenty-eight fruits of twenty-three species are presented in Figure 2. The fresh fruits were kept in cooler box; however, the dry fruits were packed in polystyrene bags before transporting to the Laboratory of Biochemistry and Biological Chemistry (LabBBC) of the University of Maroua, Cameroon.

### 2.2. Sample Preparation

Plant species and fruits were identified and authenticated at the Department of Biological Sciences of the University of Maroua (Cameroon) by Prof. Tchopsala (botanist). After this, at least 30 fruits of each species were combined in three repetitions. Then, they were washed three times with tap water, and the pulps of each fruit samples were separated from kernels and pericarps using stainless steel knives (Koch Messer, Germany). Then, pulp of each fruit samples was weighted in triplicates and dried in an air oven (Binder, USA) at 60°C for 24 hours. After that, samples were powdered using a mortar, sieved (200 *μ*m), and stored in airtight polystyrene bags, then put into opaque box at room temperature until analysis.

### 2.3. Reagents and Chemicals

Standard (98% >) such as Gallic acid, Quercetin, Catechin, Trolox, Cyanidin-3-glucoside, and also Vanillin, Trolox, ABTS, and DPPH was purchased through Sigma-Aldrich Chemical Company (Mumbai, India). Sodium carbonate, methanol, ethanol, and other solvents were obtained from Fisher commercial source (New Jersey, USA).

### 2.4. Extraction of Free and Bound Phenolic Compounds

The polyphenols were extracted as described by Laya and Koubala [13]. The amount of 0.2 g of fruit powder was mixed with 10 mL of 80% methanol and stirred for 24 hours at room temperature. After shaking, the mixture was filtered with No. 4 Whatman filter paper, and the filtrate was collected. Then, the residue was washed one more before combining the filtrates for the free phenolic fraction and kept at -4°C. For the bound phenolics, the residues were hydrolyzed with 4 mL of 2 M NaOH and incubated for 15 min in a water bath at 80°C. The mixture were cooled at room temperature and added to 2.5 mL of 2 M HCl before incubating again in a water bath for 45 min at 80°C. Then, 2.5 mL of 95% methanol was added, and the mixture was stirred for 15 min. The mixture was filtered through Whatman No. 4 filter paper before washing two times with 2.5 mL of 95% methanol. The filtrates obtained were combined for the bound phenolics and kept at -4°C until analysis.

### 2.5. Determination of Phenolic (Bound and Free) Compounds

#### 2.5.1. Total Polyphenol Content

Polyphenol (bound and free) content of the methanolic extract was determined using the Folin-Ciocalteu colorimetric method described by Singleton et al. [16]. The absorbance of phenolic compounds was measured at 745 nm using UV-VIS spectrophotometer (PRIM Light & Advanced, Germany). The calibration curve was plotted with gallic acid (0-250 *μ*g/mL; *R*^2^ = 0.9921). The results were expressed in milligram gallic acid equivalent per 100 grams edible portion (mg GAE/100 g).

#### 2.5.2. Flavonoid Content

Flavonoid (bound and free) contents of the methanolic extract were determined using the vanillin-HCl reagent as described by Brainbridge et al. [17]. The absorbance was measured at 430 nm. The calibration curve was plotted with quercetin (0-100 *μ*g/mL; *R*^2^ = 0.9924). The results were expressed in milligram quercetin equivalent per 100 grams edible portion (mg QE/100 g).

#### 2.5.3. Tannin Content

Tannin (bound and free tannin) contents of the methanolic extract were determined using aluminium chloride as described by Gaytan-Martínez et al. [18]. The tannin content was measured at 500 nm. The calibration curve was plotted with catechin (0-100 *μ*g/mL; *R*^2^ = 0.9879), and the results were expressed in milligram catechin equivalent per 100 grams edible portion (mg CE/100 g).

#### 2.5.4. Determination of Total Anthocyanins

Total anthocyanins (TA) were determined by the differential pH method as described by Lee et al. [19]. Shortly, 0.100 g of powder samples was mixed with 20 mL of acidic ethanol (HCl 0.001 N; pH 4.0), and the mixture was shaken for 1 h at room temperature. Then, the mixture was filtered through No. 4 Whatman paper filter. The filtrate was diluted with HCl/KCl (0.025 M; pH 1.0) and acetate (0.4 M; pH 4.5) buffers. After that, 200 *μ*L of diluted sample was pipetted and mixed in 1.8 mL of 25 mM HCl/KCl (pH 1.0) and 0.4 M acetic acid/sodium acetate (pH 4.5). Cyanidin-3-glucoside was used as standard, and the absorbance (A) of sample was measured at 520 and 700 nm at pH 1.0 and 4.5 buffers after 30 min of incubation, respectively, according to the following formula:

(1) $$\begin{array}{c} \text{TA}=\frac{\text{A}\times \text{Mw}\times \text{df}\times \text{V}\times 100}{\varepsilon lm} \end{array}$$

where *A* = (*A*520 − *A*700)pH 1.0 − (*A*520 − *A*700)pH4.5

(2) $$\begin{array}{c} \varepsilon =\text{Molecular extinction coefficient of the standard }\left(26900\text{ L}.{\text{cm}}^{-1}{\text{mol}}^{-1}\right)\\ \text{Mw}=\text{Molecular weigth of the standard }\left(449.2\text{ g}.\text{mol}-1\right)\\ \text{V}=\text{Volume of the sample};\text{fd}=\text{diluted factor}\\ m=\text{sample weight};\text{l}=\text{cuvette depth} \end{array}$$

The calibration curve was plotted with cyanidin-3-glucoside (0-250 *μ*g/mL; *R*^2^ = 0.9966). The results were expressed in milligram cyanidin-3-glucoside equivalent per 100 grams edible portion (mg C-3G E/100 g).

### 2.6. Minerals Determination

Mineral content in all fruits was determined using AAS wet digestion as described by Pinta et al. [20].

### 2.7. Evaluation of Antioxidant Activities of Phenolic (Free and Bound) Content

#### 2.7.1. DPPH (2.2-Diphenyl-1-Picyhydrazyl) Radical Scavenging Activity

DPPH was carried out as described by Sun et al. [21] with some modifications. Briefly, 500 *μ*L of methanolic sample (0.1 g/mL) or standard was mixed with 1500 *μ*L of 1 mM DPPH, and the mixture was stirred for 2 min before incubating for 40 min at room temperature in the dark place. The experiments were done in four repetitions. Absorbance of the mixture was measured at 517 nm. The calibration curve was plotted with trolox (0-200 *μ*g/mL; *R*^2^ = 0.9882), and the results were expressed in milligram trolox equivalent per 100 grams edible portion (mg TE/100 g).

#### 2.7.2. ABTS (2,2-Azino-Bis(3-Ethylbenzylthiozoline-6-Sulphonic Acid)) Radical Scavenging Activity

ABTS was performed as described by Re et al. [22]. Briefly, 500 *μ*L of each methanolic sample (0.1 g/mL) or standard was mixed with 1500 *μ*L of 7 mM ABTS^+^, and the mixture was shaken for 2 min before incubating for 40 min at room temperature. The experiments were done in four repetitions. Then, the absorbance was measured at 745 nm. The calibration curve was plotted with trolox (0-200 *μ*g/mL; *R*^2^ = 0.9883), and the results were expressed in milligram trolox equivalent per 100 grams edible portion (mg TE/100 g).

#### 2.7.3. FRAP (Ferric Reducing Antioxidant Power)

FRAP was determined as described by Benzie and Strain [23]. Briefly, 500 *μ*L of methanolic sample (0.1 g/mL) or standard was mixed with 1500 *μ*L of FRAP reagent (250 *μ*L of acetate buffer 0.3 M, pH 3.6, 225 *μ*L of TPTZ 0.01 M in 40 mM HCl, and 225 *μ*L of FeCl_3_ 140 mM). The experiments were done in four repetitions. Then, the mixture was shaken and incubated for 40 min at room temperature. The absorbance of the mixture was measured at 593 nm. The calibration curve was plotted with trolox (0-50 *μ*g/mL; *R*^2^ = 0.9884), and the results were expressed in milligram trolox equivalent per 100 grams edible portion (mg TE/100 g).

### 2.8. Statistical Analysis

Data were analyzed using one-way analysis of variance (ANOVA) performed by SPSS 20.0 (Inc., Chicago, IL, USA) and Graph Pad Prism 5.03, and the significance at *p* < 0.01 between all parameters was done using Tukey's tests. Pearson's correlation and principal component analysis (PCA) using XLSTAT (version 16) were done in order to establish the relationship between antioxidant activities and polyphenol content. The results are the mean of four replications expressed as mean ± standard error.

## 3. Results and Discussion

### 3.1. Phenolic Compounds of 28 Wild Edible Fruits of the Twenty-Three Species

#### 3.1.1. Variation of Total Phenolic (Free and Bound) Content of Wild Edible Fruits

The bound fraction of polyphenols of 28 fruits studied ranged between 95.58 (*A. digitata*) and 407.72 mg GAE/100 g (*B. eagyptiaca*); however, the free fraction of polyphenol content varied from 46.43 (*T. indica*) to 344.73 mg GAE/100 g (*B. eagyptiaca*) among the dry fruits (Table 2), while the bound polyphenol content ranged from 28.97 (*V. grandifolia*) to 306.04 mg GAE/100 g (*H. thebaica*) among the fresh fruits. These variations may be due to the plant species which may able to accumulate less or more the bound and the free phenolics. In comparison to the other studies, the values of the bound polyphenol content of all fruits were higher than those for miracle fruits (5.63 mg/100 g) reported by Inglett and Chen [24]. However, *H. monopetalus* (119.54 mg GAE/100 g) and *Z. mauritiana* (315.79 3 mg GAE/100 g) showed the highest and the lowest free polyphenol content (Table 2). Additionally, miracle fruit (16.95 mg/100 g) had higher content of free polyphenols than *H. monopetalus* (119.54 mg GAE/100 g) and lower than the other fresh fruits as compared to the present study. This result can be related to the methods used for quantification of the phenolics from fruits collected in different areas.

The free and bound polyphenol content evaluated for these edible fruits showed that free and bound polyphenol content varied significantly (*p* < 0.01) among fruit species (Table 2), which is in agreement with what have been observed by many authors [14, 25]. Moreover, all the dry fruits showed the highest values of bound polyphenol contents than the fresh edible fruits (Table 2). Similarly, Imeh and Khokhar [25] have reported in the literature that the amounts of bound polyphenol fraction of cultivated fruits were higher in dry fruits than in fresh fruits. Besides, Arruda et al. [26] found that the bound polyphenol content was the main polyphenol fraction in fresh *A. crassiflora* pulp. In contrast, Inglett and Chen [24] and Su et al. [14] found that the free phenolic compounds were higher in fresh miracle and litchi fruits than their bound fractions, respectively, suggesting that the free and bound polyphenol content varied according to the fruit species. In addition, Su et al. [14] found that litchi pulp contained higher content (190.69 mg GAE/100 g) of free polyphenols.

Therefore, *P. biglobosa* pulp has higher free polyphenol content (247.83 mg GAE/100 g) than that in litchi pulp as compared to the results reported by Su et al. [14]. According to its bound fraction, litchi pulp has the lowest bound polyphenol content (61.27 mg GAE/100 g) compared to that obtained in *A. senegalensis* (216.57 mg GAE/100 g). Furthermore, except *P. biglobosa* among the dry fruits and *V. paradoxa* and *X. americana* among the fresh fruits, other fruits show higher bound polyphenol content than their free fraction (Table 2). The difference for both fractions of polyphenol content may be due to the different factors such as climatic variations, ripeness at the harvest time, genetic factors, and variations in sunlight exposure [12, 27]. This higher content of bound phenolics in fruits may be benefit for the consumers because this fraction of phenolics is more active than the free [28]. Furthermore, many researchers reported that the bound phenolics can be released continuously through the gastrointestinal tract [13] and after bacterial fermentation, their bioaccesssibility and bioavailability will be high which can be used for long time for positive effects [29, 30].

#### 3.1.2. Total Flavonoid (Bound and Free) Content of Wild Edible Fruits

Total flavonoid content was also significantly (*p* < 0.01) varied among the different fruits species (Table 2). Bound flavonoid content ranged from 45.68 (*A. digitata*) to 256.87 mg QE/100 g (*B. aegyptiaca*); however, the free flavonoid content varied between 14.90 (*T. indica*) and 208.56 mg QE/100 g (*B. aegyptiaca*) among the dry fruits, while the bound flavonoid content of fresh fruits ranged from 4.27 (*P. reticulatum*) to 95.08 mg QE/100 g (*A. senegalensis*). Total free flavonoid content ranged between 19.44 (*A. senegalensis*) and 143.73 mg QE/100 g (*P. reticulatum*) (Table 2). The results showed that total bound flavonoid content of all fruits in the present study was higher than that observed in *A. crassiflora* pulp (0.28 mg QE/100 g) by Arruda et al. [26]. Except *P. biglobosa* among the dry fruits and *V. paradoxa* among the fresh fruits, other fruits had higher total bound flavonoid content than their free fraction of flavonoid content (Table 2). This result is in agreement with Arruda et al. [26], who found that *A. crassiflora* had higher total flavonoid content in its bound form. The difference in bound and free flavonoid content in plants was attributed to the genetic factors and environmental factors [12].

#### 3.1.3. Total Tannin (Bound and Free) Contents of Wild Edible Fruits

Total tannin content was significantly (*p* < 0.01) varied in respect with the dry and fresh edible fruits (Table 2). The bound fraction of tannin content ranged between 3.24 (*B. aegyptiaca*) and 57.79 mg CE/100 g of edible portion (*Z. mauritiana*) for the dry fruits, while its free forms ranged from 4.19 (*D. mespiliformis*) to 63.42 mg CE/100 g (*B. aegyptiaca*). However, the fresh fruits showed free tannin content ranging from 1.04 to 35.72 mg CE/100 g (*H. thebaica*), while the bound tannin content ranged between 1.29 (*X. america*) and 26.62 mg CE/100 g (*A. lepidofillus*) (Table 2). The results showed that *H. thebaica* has higher free tannin content than those found in litchi fruit (14.32 mg CE/100 g) analyzed by Su et al. [14].

Also, litchi fruit contains higher content of bound tannins (37.37 mg CE/100 g) than that obtained for the fresh fruits in the present study. These results demonstrate that tannins content of all fruits was higher than that found in *B. sapida* (0.372 mg CE/100 g) by Oyeleke et al. [31] during their study. These can be due to the ripeness at the time of harvest, and environmental factors such as soil type, sun exposure, rainfall, and storage conditions could be among those factors which may affect the polyphenol content in bound and free fraction of plants other than species [6]. However, the quantification of free and bound polyphenol content in wild edible fruits of the present study may provide systematic estimation of biological activities, including beneficial health effects and industrial purposes [12]. According to Li et al. [32], these wild fruits could be used as a good bioactive elements as the functional food in order to manage various diseases or use in pharmaceutical and cosmetic industries.

#### 3.1.4. Total Anthocyanin Content of Wild Edible Fruits

Found in most plant species, anthocyanin content of fruits ranged from 8.65 (*X. america*) to 98.95 *μ*g C-3G E/100 g (*P. reticulatum*) among fresh fruits, while it ranged between 14. (*Z. mauritiana*) and 168.10 *μ*g C3G E/100 g (*D. mespiliformis*) among the dry fruits (Table 2). These significant difference variations among fruits were linked with the genetic factors of the species. When compared to the work of Prvulović et al. [33], *D. mespiliformis* has higher total anthocyanin content than *P. avium,* which values ranged between 0.35 and 0.69 mg C-3G E of total anthocyanin content. Fact, the fruits with high anthocyanins content may be responsible for some biological activities including the prevention or lowering the risk of cardiovascular disease, diabetes, arthritis, and cancer [34]. These fruits can be a potential ingredient for new functional food products.

### 3.2. Antioxidant Activities of Phenolic (Free and Bound) Content of Wild Edible Fruits

Antioxidant potentials of wild fruits were evaluated by DPPH, ABTS, and FRAP methods for both bound and free fractions of phenolics. Antioxidant potentials exhibited by free and bound polyphenols of the fruits evaluated by DPPH, ABTS, and FRAP methods varied significantly (*p* < 0.01) among the species and edible forms of fruits (Table 3). The variations of the results of antioxidant capacities evaluated by the same methods were reported by many researchers [35, 36]. These variations in antioxidant activity may be due to the different modes of action of the in vitro assays used. Free polyphenols of *D. microcarpum* (120.94 mg TE/100 g) and *P. biglobosa* (784.54 mg TE/100 g) showed higher and lower DPPH radical scavenging activity for the dry fruits, respectively. However, *V. diversifolia* (40.86 mg TE/100 g) and *H. barteri* (82.71 mg TE/100 g) had the lowest and the highest values of DPPH radical scavenging among the fresh fruits. The results were in agreement with Pérez-Balladares et al. [37], who found in their study high variation of DPPH radical scavenging activity among fruits, while the bound polyphenols of dry fruits exhibited DPPH radical scavenging activity ranging between 58.88 (*D. msepiliformis*) and 559.35 mg TE/100 g (*Z. mauritiana*).

Also, DPPH radical scavenging activity of bound polyphenols from fresh fruits was ranged from 12.07 (*H. barteri*) to 302.90 mg TE/100 g (*A. senegalensis*) (Table 3). The highest DPPH radical scavenging activity was shown by *Detarium microcarpum* (120.94 mg TE/100 g) and *Diospyrus mespiliformis* (58.88 mg TE/100 g) conferring by free and bound polyphenol amount, for the dry fruits, respectively. Among fresh fruits, *H. barteri* (12.07 mg TE/100 g) and *V. diversifolia* (40.86 mg TE/100 g) showed the highest DPPH radical scavenging activity by their bound and free polyphenols, respectively. Yang et al. [12] reported that antioxidant activities of fruit species could greatly vary according to the distribution of their polyphenol form. For both fractions, bound polyphenol content of most fruits such as *D. mespiliformis*, *T. indica*, and *H. barteri* showed the strongest DPPH radical scavenging activity compared to their free fraction (Table 3). This finding is in agreement with Arruda et al. [26], who found that the bound polyphenols of *A. crassiflora* fruit showed higher antioxidant activity than free polyphenols. Similarly, Laya and Koubala [13] were found that bound polyphenols of cassava leaves showed higher DPPH radical scavenging activity than free polyphenol fraction. The highest DPPH radical savenging may linked to flavonoid content in fruits known as free radical scavengers preventing oxidative cell damage and having strong anticancer activity [38].

Furthermore, result of ABTS radical scavenging activity indicated that the free and bound polyphenols of dry fruits were stronger in *Z. mauritiana* (261.39 mg TE/100 g) and *D. mespiliformis* (86.22 mg TE/100 g), respectively (Table 3). However, *X. americana* (63.65 mg TE/100 g) and *H. barteri* (21.86 mg TE/100 g) among the fresh fruits showed the highest activity for free and bound polyphenols, respectively. Thus, ABTS radical scavenger was offered stronger activity with bound polyphenolic compounds in the dry and fresh fruits than its free fraction. Fact, phenolic acids are known as strong antioxidant compounds and can scavenge almost all oxidant molecules such as free radicals via their hydroxyl groups [10]. This may be justified by their higher content than their free amounts. Moreover, based on FRAP assays, free and bound polyphenols of *T. indica* (537.70 mg TE/g) and *Z. mauritiana* (425.53 mg TE/100 g) had a stronger antioxidant activities among the dry fruits, respectively.

In addition, the free (350.43 mg TE/100 g) and bound polyphenols (268.89 mg TE/g) of *A. senegalensis* showed the highest activity among the fresh fruits (Table 3). Compared to previous findings reported by Imeh and Khokhar [25] on apple cultivars (1.83 to 2.89 mg TE/100 g) and Kiwi (1.57 mg TE/100 g) FRAP values, their values were lower than those found in all fruits investigated in the present study. This variation in total antioxidant among fruits species was reported by Pérez-Balladares et al. [39] on various fruits. Also, the bound polyphenols of *Z. mauritiana*, *P. biglobosa*, and *D. microcarpum* among dry fruits have the highest ability to reduce ferric ions than their free forms. Except *H. barteri*, the free polyphenols of the other fresh fruits showed higher antioxidant capacity than their bound forms, suggesting that these fruits may be very important for consumers because of the longer effect of bound phenols in human body. Fact, the bound phenolics are releasing in the slower manner in the intestine and their bioactivity is higher than free phenolics. These species of wild edible fruits can be utilized for harnessing the polyphenols and antioxidant compounds and should be promoted as a source of natural antioxidant for other formulations [36]. The present results suggest that the wild fruits are a source of phenolics which could prevent oxidative DNA damage.

### 3.3. Mineral Content of Wild Edible Fruits

Minerals are important vital elements for healthy growth and development and disease prevention found in small amounts in foods. However, these substances varied in respect with the plant foods and species. The present fruits contained high amounts of P and K and relatively quantities of Ca, Mg, and Na with significant (*p* < 0.01) difference among fruits and species for macrominerals (expressed in mg/100 g in edible portion) (Table 4). The contents of Na of all fruits range from 2.01 to 54.01 in Xam and Tam, respectively. For Ca contents, the values varied between 9.74 and 57.06 in Ase and Bae fruits, respectively. The contents of K are higher compared to other macroelements that varied also significantly among fruits and species with the values ranged from 48.56 to 301.34 in Xam and Tam. Regarding the P content, the values varied between 40.32 and 118.05 in Zsp and Xam, respectively. The variations of macroelements among fruits and species found in the present study were also reported by Hegazy et al. [40] when they evaluated the minerals composition of some wild edible fruits in the three study species from Middle East of Egypt. In comparison with the fruits form, microelements (expressed in mg/100 g edible portion) such as Mn, Cu, and Se are higher in fresh than dry fruits (Table 4). However, the contents of Mn ranged from 0.45 to 4.56 in Hba and Ale, respectively, while Fe contents ranged from 0.99 to 2.77 in Ada and Zma, respectively. Fruits Dmi (0.13) and Boe (1.13) had the highest and the lowest values of Cu in fruits, respectively. The present study showed that Cu is in low amount among microelements compared to others (Table 4).

Zn contents found at higher amount among fruits varied between 0.68 and 10.26 in Dme and Ada, respectively. These significant variations of microelements among fruits and species were similar with the results obtained by Sibiya et al. [41] in their study of mineral composition of selected indigenous wild southern African fruits. Our fruits are rich in microelements (Fe, Zn and Cu) compared to the contents (0.00-0.27 mg/100 g) found in four fruit jams reported by Naeem et al. [42]. This result could be due to specie, variety, location, soil, and climatic conditions. In fact, Paunović et al. [43] found that minerals in black mulberry fruit varied significantly among 3 locations. Se is an essential mineral that have high antioxidant activity found with high amount in Boe which can be considered a source of Se which will be benefit for fruit consumers.

### 3.4. Correlation between Phenolic Compounds and Antioxidant Activities

The antioxidant activities evaluated in the present study are highly and positively correlated with total polyphenols, flavonoids and tannins content (Table 5). Various findings reported relationships between polyphenols content and antioxidant activity of fruits [24, 44]. Similarly, and Surveswaran et al. [45] reported significant and positive linear correlations between total antioxidant capacities and phenolic contents.

In the present study, taken apart each fraction of polyphenol content, it is observed that bound polyphenol content shows a significant and positive relationship with ABTS (*r* = 0.690, *p* < 0.01) and FRAP (*r* = 0.747, *p* < 0.01). Also, significant and positive relationships between antioxidant activities and bound polyphenol content in these fruits were similar with other studies on some exotic fruits [23, 38]. However, no association is shown by bound forms of polyphenolic compounds performed through the DPPH assay.

In fact, according to the mechanisms, based on the types of the induced compound antioxidant activities, ABTS radical scavenging is conferred by both lipophilic and hydrophilic compounds, while DPPH radical capturing is induced by hydrophilic compound [46]. These results suggest that lipophilic bound polyphenolic compounds were the major contributors to the antioxidant activity of the fruits evaluated by the two radical scavenging methods. However, free polyphenol content shows higher significant correlative values with ABTS (*r* = 0.889; *p* < 0.01) and DPPH (*r* = 0.784; *p* < 0.01), while these show average relationships with FRAP (*r* = 0.664; *p* < 0.01). Moreover, free polyphenol content shows stronger correlation with ABTS (*r* = 0.889; *p* < 0.01) and DPPH (*r* = 0.784; *p* < 0.01) than the bound forms; however, the contrary is shown with FRAP value. High correlation observed in the present study is not in agreement with Imeh and Khokhar [25], who found a weak correlation (*r* = 0.518) between total polyphenols and total antioxidant activity of 16 fruits.

According to the type of polyphenolic compounds and to the DPPH scavenging activity, the present results show that most of the free polyphenolic compounds of fruits may be hydrophilic than that in the bound forms. Flavonoids, another antioxidant compounds found in large amounts in plants, show a significant and positive correlation between its two fraction (bound and free) contents (Table 5). Bound flavonoid content is significantly and positively associated with DPPH (*r* = 0.694, *p* < 0.01) and FRAP (*r* = 0.767, *p* < 0.01). The positive association is shown between free fraction content of flavonoids with ABTS (*r* = 0.720, *p* < 0.01), DPPH (*r* = 0.534, *p* < 0.01), and FRAP (*r* = 0.620, *p* < 0.01). Additionally, bound flavonoids show higher associative values than their free forms performed with the three methods (Table 5). Throughout the result, free and bound flavonoid content contributes to the DPPH radical scavenging capacity and FRAP, while bound forms contribute more than its free forms to ABTS radical scavenging activity.

The present findings are in accordance with the reports of those who found that flavonoids were the main contributors to antioxidant activities from *A. crassiflora* [26] and grape fruits [46] due to the presence of double bonds in their C-rings, which increase their nucleophilic power. Moreover, tannin content with its bound fraction content shows only a significant and positive relationship with FRAP (*r* = 0.603, *p* < 0.01). The free polyphenol fraction is lowly and positively related to ABTS (*r* = 0.408, *p* < 0.01), DPPH (*r* = 0.527, *p* < 0.01), and FRAP (*r* = 0.382, *p* < 0.01) antioxidant properties. The results also revealed that free tannin content contributes to ABTS and DPPH radical scavenging activities, while bound ones mostly confer FRAP activity (Table 5). The different antioxidant capacity assays evaluated in the present study show a positive relationship between one form to another which vary significantly according to the antioxidant assays (Table 5). Concerning to the contribution of the two forms of polyphenol content evaluated in this work, the results lead to know that bound polyphenol content contributes to the antioxidant activities of the free forms performed by the ABTS method (*r* = 0.287, *p* < 0.01). Additionally, free polyphenol content contributes to the bound polyphenol antioxidant activities through ABTS (*r* = 0.452, *p* < 0.01) and DPPH (*r* = 0.493, *p* < 0.01). However, free fractions of flavonoids show strong contribution to the antioxidant properties of bounds performed with ABTS (*r* = 0.868, *p* < 0.01) and DPPH (*r* = 0.729, *p* < 0.01). Moreover, free tannin content contributes to the bound forms with the FRAP assay (*r* = 0.279, *p* < 0.01).

The contribution of one form of polyphenolic compounds to another in the antioxidant capacities of the wild fruits found in the present study is in agreement with Arruda et al. [26], who observed that antioxidant activity of polyphenol compounds is affected by intermolecular interactions, which can be either synergistic or antagonistic, depending on the conditions and compounds under study. Once more, as reported by Arruda et al. [26], the contribution of phenolic content to antioxidant activity in a food will therefore depend on its concentration and chemical features, matrix composition, and medium conditions. The present study suggests clearly that the wild fruits can offer greater potential sources of natural antioxidants since no previous study had directly examined the contributions of bound and free polyphenols in the antioxidant capacity.

### 3.5. Principal Component Analysis (PCA)

**Figure 3:**
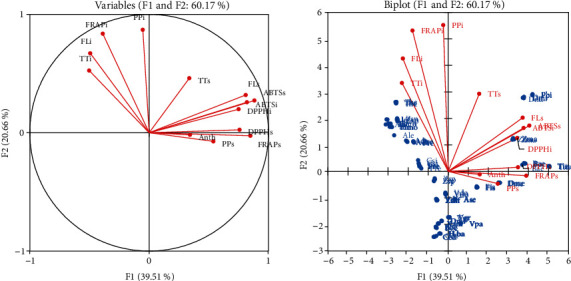
Principal component analysis (PCA) means showing the relationship among total polyphenols (free and bound), total flavonoids (free and bound), total tannins (free and bound), and antioxidant activities. (a) Correlation between variables and factors and (b) biplot of fruit distribution according to their polyphenolic contents and antioxidant activities. PPi: bound polyphenols; FLi: bound flavonoids; TTi: bound tannins; ABTSi: ABTS activity of the bound phenolic compounds; DPPHi: DPPH activity of the bound phenolic compounds; FRAPi: FRAP activity of the bound phenolic compounds; PPs: free polyphenols; FLs: free flavonoids; TTs: free tannins; ABTSs: ABTS activity of the free phenolic compounds; DPPHs: DPPH activity of the free phenolic compounds; FRAPs: FRAP of the free phenolic compounds; TA: total anthocyanins. Blue color indicates different samples.

To further discover the contribution of each fruit according its phenolic contents in the antioxidant potentials, PCA showed clearly the separation between bound and free polyphenol content (Figure 3).  Figure 3(a) shows that the polyphenolic compounds and antioxidant activities quantified in the fruits were reduced into two main components (F1 and F2) by the principal component analysis. F1 and F2 explain 60.17% of total data variance, with F1 alone accounting for 39.51% of the observed variations. The variables which mainly contributed positively (F loading >0.50) to F1 are bound flavonoids, bound polyphenols, insoluble forms of antioxidants conferred by FRAP. However, F2 accounts for 20.66% of the observed variations were free flavonoids, ABTS, and DPPH which contribute positively (F loading > 0.75). Therefore, the main fruits species which have contributed mostly to the antioxidant capacities with their polyphenol content are divided into two groups according to F1 and F2 components (Figure 3(b)). However, *A. lepilidofilus*, *H. monopetalus*,and the two forms of *Z. spina-christi* and *P. reticulatum* are the most contributors to the F1 component, while, *P. biglobosa*, *T. indica*, *B. aegyptiaca*, *D. microcarpum*, and the two edible forms of *Z. mauritiana* for F2.

## 4. Conclusions

The aims of the present study is to investigate for the first time the polyphenol compound (bound and free) content and their antioxidant activities of the most consumed wild edible fruits native of the Far North Region of Cameroon. Polyphenolic compounds and antioxidant activities of these fruits were quantified for their free and bound fractions. Except *P. reticulatum* and *A. digitata*, other dry fruits contain higher amounts of bound polyphenols than their free forms content. Bound polyphenolic compounds of *P. reticulatum* show strong ABTS radical scavenging activity, while DPPH radical scavenging activity and FRAP values were both recorded in *T. indica* among dry fruits. However, the highest DPPH, ABTS, and FRAP activities among fresh fruits were shown by *V. diversifolia*, *X. americana*, and *C. edulis*, respectively, for free polyphenol content. Bound polyphenols of *H. monopetalus*, *H. barteri*, and *C. sinensis* showed the highest DPPH and ABTS radical scavenging activity and FRAP values. Furthermore, significant and highly positive correlation among antioxidant activities and phenolic content is established. The present study revealed that the wild edible fruits are rich in free and bound phenolic compounds as sources of antioxidants with high antioxidant activities. The mineral content analysis indicates that wild fruits are rich in valuable macro- and trace elements. Also, correlation between phenolic compounds and antioxidant capacities showed that phenolics may be responsible of the biological activities when fruits are consumed. Thus, consumption of these wild fruits can offer benefits for consumer's health through the supply of natural bioactive compounds which are associated to the prevention of diseases. However, biological activities (antidiabetic and antiobesity) of phenolics of these fruits will be investigated in the future.

## Figures and Tables

**Table 1:** Ethnopharmacological effects of different wild edible fruits.

Botanical names	Family	Morphology	Ethnopharmacology effects
*Ziziphus mauritiana*	Rhamnaceae	Shrub	Antidysentery, against jaundice, Amibiae
*Ziziphus spina-christi*		Tree	Against jaundice, antibacterial
*Carissa edulis*	Boraginaceae	Shrub	Abdominal pain reliever, against jaundice and constipation
*Tamarindus indica*	Caesalpiniaceae	Tree	Antihypertension, against constipation and indigestion, antipyretic, aphrodisiac
*Detarium microcarpum*		Tree	Antianorexia, against kidney problem, antiamenorrhea
*Parkia biglobosa*	Mimosaceae	Tree	Against constipation and cough, antibacterial
*Balanites aegyptiaca*	Balanitaceae	Tree	Against constipation and kidney failure, antidysentery, against undigestion, aphrodisiac, abdominal pain reliever, antileprotic, antidiabetic
*Phoenix reticulatum*	Arecaceae	Tree	Antidiarrheal, abdominal pain reliever
*Hyphaene thebaica*		Tree	Antihypertension, against constipation, indigestion and asthenia
*Borassus eathiopium*		Tree	Against constipation and anemia
*Vitex grandifolia*	Verbenaceae	Shrub	Abdominal pain reliever
*Vitex doniana*		Shrub	Againts asthenia and constipation, abdominal pain reliever, antiemetic
*Vitex diversifolia*		Shrub	Against cough and anorexia
*Vitellaria paradoxa*	Sapotaceae	Tree	Antidiarrheal, against anemia
*Ximenia Americana*	Olacaceae	Shrub	Against constipation and anemia, undigestion
*Afrostyrax lepidophyllus*	Huaceae	Shrub	Antibacterial, antistomachic, preservative, antimeasles and against mumps
*Haematostaphis Barteri*	Anacardiaceae	Tree	Anemia, antigastritis, headache
*Ficus dicranostyla*	Moraceae	Tree	Antianorexia, against jaundice
*Annona senegalensis*	Annonaceae	Shrub	Against constipation
*Hexabolus monopetalus*		Tree	Against kidney problems, anti-inflammatory
*Adansonia digitata*	Bombacaceae	Tree	Antiagalactia, against asthenia
*Diospyros mespiliformis*	Ebenaceae	Tree	Antidiarrheal, against jaundice
*Cordia sinensis*	Apocynaceae	Shrub	Antianemia, antiscurvy

**Table 2:** Polyphenolic (bound and free) compounds (mg/100 g EP) of twenty-eight wild edible fruits of twenty-three species.

Fruits		Total polyphenols (mg GAE/100 g)		Total flavonoids (mg QE/100 g)		Total tannins (mg CE/100 g)		Anthocyanins (mg C3GE/100 g)
		Free fraction	Bound fraction	Free fraction	Bound fraction	Free fraction	Bound fraction	
Dry	Zma	94.00 ± 0.15^n^	142.41 ± 0.38^k^	26.42 ± 0.07^l^	107.20 ± 0.11^e^	8.28 ± 0.15^g^	4.76 ± 0.08^f^	14.01 ± 0.55^ij^
	Pbi	247.83 ± 0.25^f^	270.79 ± 0.21^d^	189.88 ± 0.33^b^	159.37 ± 0.11^c^	16.05 ± 0.06^e^	57.79 ± 0.16^a^	129.38 ± 0.30^b^
	Dmi	146.43 ± 0.10^l^	194.96 ± 0.23^h^	116.46 ± 0.04^e^	135.73 ± 0.34^c^	6.26 ± 0.06^fg^	41.18 ± 0.03^b^	168.10 ± 0.53^a^
	Bae	344.73 ± 0.33^b^	407.72 ± 0.12^a^	208.56 ± 0.64^a^	256.87 ± 0.43^a^	63.42 ± 0.95^a^	3.24 ± 0.63^f^	164.18 ± 0.64^a^
	Pre	309.59 ± 0.22^cd^	159.05 ± 0.60^jk^	203.76 ± 0.93^a^	89.59 ± 0.39^g^	27.87 ± 0.62^c^	10.28 ± 0.76^e^	167.98 ± 0.57^a^
	Ada	296.02 ± 0.36^d^	95.58 ± 0.04^m^	187.34 ± 0.82^b^	45.68 ± 0.87^j^	42.35 ± 0.43^b^	22.31 ± 0.06^d^	86.45 ± 0.56^d^
	Zsp	169.12 ± 0.81^k^	271.40 ± 0.16^d^	98.65 ± 0.03^f^	127.56 ± 0.93^d^	11.56 ± 0.34^ef^	32.64 ± 0.53^cd^	100.76 ± 0.94^c^
	The	238.54 ± 0.65^f^	332.45 ± 0.43^b^	176.23 ± 0.63^c^	214.62 ± 0.21^b^	20.51 ± 0.67^d^	52.78 ± 0.68^ab^	126.65 ± 0.32^b^
	Dme	56.43 ± 0.06^o^	178.71 ± 0.08^i^	22.48 ± 0.02^lm^	104.70 ± 0.05^ef^	4.19 ± 0.02^gh^	4.29 ± 0.07^f^	39.76 ± 0.75^g^
	Tam	46.43 ± 0.10^o^	208.03 ± 0.23^gh^	14.90 ± 0.07^n^	135.23 ± 0.08^c^	12.51 ± 0.02^ef^	56.83 ± 0.08^a^	23.96 ± 0.56^h^

Fresh	Vdi	138.14 ± 0.09^l^	214.26 ± 0.01^g^	19.46 ± 0.07^f^	29.81 ± 0.11^j^	12.47 ± 0.04^ef^	13.17 ± 0.03^e^	94.15 ± 0.24^cd^
	Vpa	272.35 ± 0.18^e^	230.21 ± 0.08^ef^	40.71 ± 0.08^j^	7.53 ± 0.02^lm^	21.04 ± 0.03^d^	8.10 ± 0.02^ef^	20.40 ± 0.57^hi^
	Hba	214.25 ± 0.01^hi^	137.54 ± 0.02^k^	35.66 ± 0.00^k^	26.40 ± 0.01^jl^	1.04 ± 0.00^i^	8.73 ± 0.01^ef^	19.15 ± 0.90^hi^
	Ase	246.02 ± 0.12^f^	216.57 ± 0.12^gf^	19.44 ± 0.05^mn^	95.08 ± 0.09^f^	17.01 ± 0.04^ed^	7.39 ± 0.04^ef^	45.39 ± 0.60^g^
	Xam	252.69 ± 0.06^f^	223.39 ± 0.04^f^	63.54 ± 0.04^hi^	17.17 ± 0.03^l^	4.84 ± 0.01^gh^	1.29 ± 0.01^g^	8.65 ± 0.15^j^
	Pre	209.30 ± 0.31^i^	149.15 ± 0.37^jk^	143.73 ± 0.45^d^	4.27 ± 0.03^m^	29.56 ± 0.23^c^	3.46 ± 0.52^f^	98.95 ± 0.86^c^
	Afl	189.48 ± 0.44^j^	229.75 ± 0.02^ef^	27.45 ± 0.65^kl^	89.34 ± 0.34^g^	12.36 ± 0.42^ef^	26.62 ± 0.12^c^	79.55 ± 0.10^df^
	Vdo	184.07 ± 0.11^j^	169.36 ± 0.38^ij^	56.46 ± 0.18^i^	28.92 ± 0.41^j^	9.35 ± 0.21^gf^	2.67 ± 0.38^fg^	48.59 ± 0.37^g^
	Fid	230.75 ± 0.25^g^	203.32 ± 0.12^h^	77.36 ± 0.82^g^	68.46 ± 0.56^i^	14.24 ± 0.25^ef^	7.35 ± 0.08^ef^	49.76 ± 0.83^g^
	Csi	292.30 ± 0.17^d^	234.06 ± 0.01^e^	43.93 ± 0.52^j^	12.45 ± 0.73^kl^	22.40 ± 0.27^dc^	1.95 ± 0.46^fg^	15.84 ± 0.30^i^
	Vgr	202.60 ± 0.42^i^	28.97 ± 0.15^n^	77.23 ± 0.30^g^	9.34 ± 0.37^l^	16.20 ± 0.52^e^	3.85 ± 0.74^f^	11.35 ± 0.77^ij^
	Boe	168.62 ± 0.12^k^	212.18 ± 0.32^g^	23.42 ± 0.76^lm^	79.56 ± 0.45^hi^	6.15 ± 0.65^g^	13.16 ± 0.86^e^	12.01 ± 0.84^ij^
	Dme	269.50 ± 0.31^e^	295.18 ± 0.16^c^	79.43 ± 0.34^g^	14.67 ± 0.25^k^	17.42 ± 0.23^ed^	11.98 ± 0.67^e^	79.97 ± 0.43^df^
	Hmo	119.54 ± 0.43^m^	143.87 ± 0.52^k^	67.34 ± 0.52^h^	8.21 ± 0.17^l^	13.14 ± 0.39^ef^	2.35 ± 0.07^b^	15.31 ± 0.41^i^
	Hte	378.39 ± 0.09^a^	306.04 ± 0.42^c^	82.36 ± 0.10^g^	135.37 ± 0.61^c^	35.72 ± 0.50^bc^	14.32 ± 0.35^e^	73.67 ± 0.48^e^
	Zsp	229.50 ± 0.13^g^	121.89 ± 0.34^l^	93.03 ± 0.45^fg^	74.30 ± 0.12^hi^	12.23 ± 0.65^ef^	4.56 ± 0.29^f^	67.85 ± 0.12^e^
	Zma	315.79 ± 0.23^c^	270.46 ± 0.17^d^	76.35 ± 0.17^g^	32.18 ± 0.72^j^	6.89 ± 0.26^fg^	3.51 ± 0.18^f^	25.17 ± 0.16^h^
	Ced	218.12 ± 0.14^h^	154.90 ± 0.04^j^	69.45 ± 0.23^gh^	25.86 ± 0.24^jl^	17.38 ± 0.60^ed^	8.23 ± 0.54^ef^	19.89 ± 0.76^hi^

Dmi*: Detarium microcarpum*; Pbi*: Parkia biglobosa*; Zma*: Ziziphus mauritiana*; Bae*: Balanites eagyptiaca*; Dme*: Diospyrus mespiliformis*; Tam*: Tamarindus indica;* Ada*: Adansonia digitata*; Zsp*: Ziziphus spina-christi*; Pre*: Phoenix reclinatum;* Hts*: Hyphaene thebaica;* Vdi*: Vitex diversifolia;* Vpa*: Vitellaria paradoxa*; Hba*: Haematostaphis barteri*; Ase*: Annona senegalensis*; Xam*: Ximenia americana*; Csi*: Cordia sinensis*; Ced*: Carissa edulis*; Fid*: Ficus dicranostyla*; Ale*: Afrostyrax lepidophyllus*; Hmo*: Hexabolus monopetalus*; *Boe: Borassus aethiopium.* Values are the means ± SE with four replicates per specie. mgEGA/100 g: milligrams equivalent gallic acid per 100 grams edible portion; mgEQ/100 g: milligram equivalent quercetin per 100 grams edible portion; mgECat/100 g: milligram equivalent catechin per 100 grams edible portion; mg C3GE/100 g: milligram equivalent cyanidin-3-glucoside per 100 grams edible portion. In the same column, values followed by different superscript letters are significantly different (*p* < 0.01).

**Table 3:** Antioxidant activities of polyphenolic (free and bound) compounds (mg TE/100 g EP) of twenty-eight wild edible fruits.

Fruits		DPPH		ABTS		FRAP	
		Free fraction	Bound fraction	Free fraction	Bound fraction	Free fraction	Bound fraction
Dry	Zma	172.68 ± 0.10^g^	559.35 ± 0.54^a^	261.39 ± 0.44^ij^	344.40 ± 0.54^a^	233.58 ± 0.26^h^	425.53 ± 0.10^d^
	Pbi	784.54 ± 0.55^a^	239.43 ± 0.22^c^	379.09 ± 0.82^e^	286.61 ± 0.44^b^	225.77 ± 0.23^h^	322.43 ± 0.13^fg^
	Dmi	120.94 ± 0.22^j^	73.64 ± 0.58^p^	700.32 ± 0.82^b^	263.45 ± 0.30^c^	132.88 ± 0.26^l^	313.41 ± 0.21^g^
	Bae	34.56 ± 0.56^u^	245.78 ± 0.78^e^	167.34 ± 0.87^m^	89.03 ± 0.45^i^	172.12 ± 0.76^jk^	193.60 ± 0.12^m^
	Pre	78.56 ± 0.23^m^	64.67 ± 0.09^q^	325.98 ± 0.45^f^	36.04 ± 0.56^t^	387.65 ± 0.73^d^	332.76 ± 0.02^f^
	Ada	128.65 ± 0.69^i^	76.43 ± 0.39^o^	146.77 ± 0.36^o^	53.72 ± 0.67^r^	98.63 ± 0.37^o^	196.64 ± 0.56^m^
	Zsp	254.98 ± 0.45^e^	98.45 ± 0.84^k^	45.67 ± 0.56^a^	167.82 ± 0.63^f^	345.07 ± 0.13^e^	132.75 ± 0.63^o^
	The	156.67 ± 0.78^f^	128.53 ± 0.34^i^	66.43 ± 0.72^u^	48.21 ± 0.16^s^	452.64 ± 0.52^b^	276.87 ± 0.46^i^
	Dme	405.76 ± 0.42^d^	158.88 ± 0.07^g^	265.97 ± 0.28^h^	86.22 ± 0.10^j^	313.62 ± 0.25^f^	52.99 ± 0.07^q^
	Tam	740.45 ± 0.15^b^	58.49 ± 0.21^r^	463.10 ± 0.40^d^	105.26 ± 0.01^h^	537.70 ± 0.43^a^	653.12 ± 0.08^a^

Fresh	Vdi	40.86 ± 0.01^t^	58.24 ± 0.09^b^	96.06 ± 0.20^st^	74.71 ± 0.15^m^	276.31 ± 0.04^g^	451.09 ± 0.05^c^
	Vpa	49.39 ± 0.10^s^	80.90 ± 0.17^mn^	103.06 ± 0.18^q^	62.67 ± 0.06^o^	174.05 ± 0.31^jk^	223.86 ± 0.10^kl^
	Hba	62.71 ± 0.08^q^	76.07 ± 0.00^n^	67.34 ± 0.05^u^	21.86 ± 0.01^v^	79.41 ± 0.03^p^	113.37 ± 0.01^p^
	Ase	48.04 ± 0.13^bc^	92.90 ± 0.20^l^	107.45 ± 0.27^q^	103.08 ± 0.15^h^	350.43 ± 0.21^e^	468.89 ± 0.16^c^
	Xam	68.56 ± 0.10^p^	56.36 ± 0.15^r^	63.65 ± 0.10^v^	59.93 ± 0.17^p^	185.66 ± 0.04^j^	237.06 ± 0.03^k^
	Pre	53.75 ± 0.46^r^	83.48 ± 0.27^m^	187.67 ± 0.35^l^	209.30 ± 0.31^d^	349.15 ± 0.37^e^	143.73 ± 0.45^o^
	Afl	87.23 ± 0.16^fg^	96.48 ± 0.67^kl^	167.43 ± 0.23^m^	89.48 ± 0.44^i^	129.75 ± 0.02^l^	327.45 ± 0.65^f^
	Vdo	77.37 ± 0.27^no^	92.35 ± 0.45^l^	987.23 ± 0.19^a^	84.07 ± 0.11^k^	69.36 ± 0.38^pq^	256.46 ± 0.18^j^
	Fid	138.03 ± 0.47^h^	73.72 ± 0.17^p^	543.36 ± 0.82^c^	30.75 ± 0.25^u^	103.32 ± 0.12^o^	17.36 ± 0.82^r^
	Csi	76.34 ± 0.69^o^	143.46 ± 0.45^h^	92.34 ± 0.11^t^	78.30 ± 0.17^l^	34.06 ± 0.01^r^	514.93 ± 0.52^b^
	Vgr	84.56 ± 0.26^l^	203.38 ± 0.23^f^	314.50 ± 0.35^g^	102.60 ± 0.42^h^	128.97 ± 0.15^lm^	177.23 ± 0.30^n^
	Boe	97.45 ± 0.62^k^	133.83 ± 0.34^i^	245.48 ± 0.62^k^	68.62 ± 0.12^n^	112.18 ± 0.32^n^	223.42 ± 0.76^kl^
	Dme	634.03 ± 0.47^c^	279.45 ± 0.22^d^	243.87 ± 0.72^k^	169.50 ± 0.31^e^	95.18 ± 0.16^o^	179.43 ± 0.34^n^
	Hmo	52.65 ± 0.12^rs^	35.46 ± 0.34^m^	152.87 ± 0.56^n^	119.54 ± 0.43^g^	43.87 ± 0.52^r^	67.34 ± 0.52^q^
	The	66.27 ± 0.36^p^	76.42 ± 0.42^o^	65.67 ± 0.83^uv^	78.39 ± 0.09^l^	206.04 ± 0.42^i^	182.36 ± 0.10^mn^
	Zsp	52.02 ± 0.47^c^	73.63 ± 0.74^p^	98.35 ± 0.62^r^	89.50 ± 0.13^i^	121.89 ± 0.34^m^	293.03 ± 0.45^gh^
	Zma	78.37 ± 0.49^mn^	98.72 ± 0.98^k^	256.87 ± 0.54^j^	115.79 ± 0.23^gh^	70.46 ± 0.17^pq^	376.35 ± 0.17^e^
	Ced	227.48 ± 0.23^i^	378.81 ± 0.37^b^	123.78 ± 0.52^p^	118.12 ± 0.14^g^	413.90 ± 0.04^c^	169.45 ± 0.23^n^

Dmi*: Detarium microcarpum;* Pbi*: Parkia biglobosa;* Zma*: Ziziphus mauritiana;* Bae*: Balanites eagyptiaca;* Dme*: Diospyrus mespiliformis;* Tam*: Tamarindus indica;* Ada*: Adansonia digitata;* Zsp*: Ziziphus spina-christi;* Pre*: Phoenix reclinatum;* Hts*: Hyphaene thebaica;* Vdi*: Vitex diversifolia;* Vpa*: Vitellaria paradoxa;* Hba*: Haematostaphis barteri;* Ase*: Annona senegalensis;* Xam*: Ximenia americana;* Csi*: Cordia sinensis;* Ced*: Carissa edulis;* Fid*: Ficus dicranostyla;* Ale*: Afrostyrax lepidophyllus;* Hmo*: Hexabolus monopetalus; Boe: Borassus aethiopium.* Values are the means ± SE with four replicates per specie, and results were expressed in milligram equivalent trolox per 100 grams edible portion (mgET/100 g EP). In the same column, values followed by different superscript letters are significantly different (*p* < 0.01).

**Table 4:** Mineral content in seventeen wild fruits (values are expressed in mg/100 g in edible portion).

	Macrominerals (mg/100 g)					Microminerals				
	Na	Ca	Mg	K	P	Mn	Fe	Cu	Se	Zn
Zma	48.34 ± 0.01^a^	36.24 ± 0.01^c^	9.21 ± 0.04^f^	158.12 ± 0.05^c^	78.02 ± 0.01^d^	3.01 ± 0.01^c^	2.77 ± 0.02^a^	0.25 ± 0.01^g^	0.93 ± 0.01^c^	5.01 ± 0.01^d^
Pbi	30.65 ± 0.05^b^	24.65 ± 0.02^e^	57.41 ± 0.01^b^	96.02 ± 0.15^e^	54.36 ± 0.03^f^	2.15 ± 0.02^d^	1.45 ± 0.01^de^	0.58 ± 0.02^e^	0.23 ± 0.00^i^	7.10 ± 0.03^c^
Dmi	5.22 ± 0.01^f^	15.04 ± 0.04^f^	32.25 ± 0.04^c^	74.21 ± 0.24^g^	42.89 ± 0.05^g^	0.96 ± 0.06^g^	1.63 ± 0.01^d^	0.13 ± 0.01^h^	0.32 ± 0.01^i^	3.13 ± 0.04^e^
Bae	18.21 ± 0.00^d^	57.06 ± 0.01^a^	68.26 ± 0.00^a^	116.01 ± 0.01^d^	102.58 ± 0.01^b^	1.45 ± 0.05^e^	2.85 ± 0.10^a^	0.86 ± 0.05^c^	0.59 ± 0.03^g^	6.30 ± 0.01^b^
Pre	25.14 ± 0.12^c^	32.01 ± 0.02^c^	16.45 ± 0.01^e^	59.25 ± 0.06^h^	69.01 ± 0.02^de^	2.50 ± 0.03^d^	1.25 ± 0.15^e^	0.45 ± 0.01^f^	0.51 ± 0.03^g^	0.98 ± 0.02^i^
Ada	35.24 ± 0.01^b^	45.15 ± 0.06^b^	21.05 ± 0.01^e^	126.21 ± 0.04^d^	89.34 ± 0.01^c^	1.32 ± 0.01^e^	0.99 ± 0.00^f^	0.26 ± 0.00^g^	0.78 ± 0.02^d^	10.26 ± 0.01^a^
Zsp	31.01 ± 0.05^b^	28.35 ± 0.01^cd^	5.24 ± 0.02^g^	204.09 ± 0.35^b^	118.05 ± 0.03^a^	2.78 ± 0.02^d^	2.02 ± 0.11^b^	0.69 ± 0.01^d^	0.82 ± 0.00^cd^	1.88 ± 0.03^g^
Dme	9.54 ± 0.14^e^	52.02 ± 0.03^a^	11.04 ± 0.04^f^	102.01 ± 0.08^e^	95.21 ± 0.12^b^	3.28 ± 0.20^b^	1.87 ± 0.00^c^	1.04 ± 0.00^bc^	0.45 ± 0.01^h^	0.68 ± 0.00^j^
Tam	54.01 ± 0.25^a^	25.24 ± 0.01^d^	28.67 ± 0.03^d^	301.34 ± 0.01^a^	107.35 ± 0.06^b^	1.14 ± 0.15^e^	2.54 ± 0.03^a^	0.53 ± 0.02^e^	0.42 ± 0.02^h^	4.36 ± 0.03^e^
*Vdi*	3.28 ± 0.05^f^	14.25 ± 0.04^f^	20.09 ± 0.07^e^	88.32 ± 0.06^f^	59.29 ± 0.04^ef^	0.89 ± 0.05^h^	1.02 ± 0.01^f^	0.44 ± 0.01^f^	0.65 ± 0.01^f^	1.30 ± 0.01^h^
*Vpa*	11.94 ± 0.01^e^	43.05 ± 0.01^b^	35.02 ± 0.05^c^	124.95 ± 0.02^d^	65.09 ± 0.05^e^	4.36 ± 0.36^a^	2.09 ± 0.01^b^	0.61 ± 0.03^de^	1.06 ± 0.02^b^	3.01 ± 0.02^e^
*Hba*	18.02 ± 0.25^d^	34.09 ± 0.11^c^	4.57 ± 0.01^g^	90.18 ± 0.01^ef^	77.68 ± 0.01^d^	0.45 ± 0.03^i^	1.52 ± 0.02^d^	0.70 ± 0.01^d^	0.89 ± 0.01^b^	9.32 ± 0.01^b^
*Ase*	22.35 ± 0.00^cd^	9.74 ± 0.08^g^	15.06 ± 0.13^ef^	68.29 ± 0.02^gh^	88.14 ± 0.03^c^	1.22 ± 0.01^e^	2.04 ± 0.01^bc^	0.91 ± 0.03^c^	0.76 ± 0.02^ef^	1.65 ± 0.00^g^
*Xam*	2.01 ± 0.08^g^	26.08 ± 0.02^de^	6.25 ± 0.07^g^	48.56 ± 0.04^i^	40.32 ± 0.02^g^	2.01 ± 0.03^d^	1.98 ± 0.02^c^	2.01 ± 0.03^a^	1.15 ± 0.01^a^	2.69 ± 0.01^f^
*Ale*	12.58 ± 0.02^e^	33.07 ± 0.06^c^	19.56 ± 0.08^e^	63.32 ± 0.02^h^	57.25 ± 0.01^f^	4.56 ± 0.04^a^	2.14 ± 0.00^b^	0.79 ± 0.01^cd^	0.98 ± 0.08^b^	3.99 ± 0.02^e^
*Boe*	15.23 ± 0.03^de^	27.32 ± 0.05^d^	56.09 ± 0.04^b^	67.02 ± 0.03^gh^	96.21 ± 0.00^b^	2.56 ± 0.00^d^	1.34 ± 0.10^de^	1.13 ± 0.01^b^	1.28 ± 0.01^a^	2.32 ± 0.01^f^
*Hmo*	8.65 ± 0.25^ef^	21.39 ± 0.01^e^	17.05 ± 0.05^e^	101.05 ± 0.06^e^	72.37 ± 0.03^d^	3.65 ± 0.02^b^	1.90 ± 0.04^c^	0.40 ± 0.03^f^	0.79 ± 0.02^cd^	1.82 ± 0.01^g^

Dmi*: Detarium microcarpum;* Pbi*: Parkia biglobosa;* Zma*: Ziziphus mauritiana;* Bae*: Balanites eagyptiaca;* Dme*: Diospyrus mespiliformis;* Tam*: Tamarindus indica;* Ada*: Adansonia digitata;* Zsp*: Ziziphus spina-christi;* Pre*: Phoenix reclinatum;* Vdi*: Vitex diversifolia;* Vpa*: Vitellaria paradoxa;* Hba*: Haematostaphis barteri;* Ase*: Annona senegalensis;* Xam*: Ximenia americana;* Ale*: Afrostyrax lepidophyllus;* Hmo*: Hexabolus monopetalus; Boe: Borassus aethiopium.* Values are the means ± SE with three replicates. In the same column, values followed by different superscript letters are significantly different (*p* < 0.01).

**Table 5:** Pearson's correlation between polyphenolic compounds and antioxidant properties of twenty-eight wild edible fruits.

	PPi	FLi	TTi	ABTSi	DPPHi	FRAPi	PPs	FLs	TTs	ABTSs	DPPHs	FRAPs	TA
PPi	1	.540^∗∗^	.375^∗∗^	.690^∗^	.154	.747^∗∗^	-.149	.150	.379^∗∗^	.287^∗∗^	-.111	-.147	-.087
FLi		1	.507^∗∗^	-.277^∗∗^	.694^∗∗^	.767^∗∗^	-.250^∗∗^	-.236^∗^	.027	-.280^∗∗^	-.175	-.290^∗∗^	-.180
TTi			1	-.157	-.104	.603^∗∗^	-.106	-.055	-.044	-.370^∗∗^	-.433^∗∗^	-.449^∗∗^	-.261^∗∗^
ABTSi				1	.906^∗∗^	-.140	.452^∗∗^	.868^∗∗^	.128	.788^∗∗^	.386^∗∗^	.544^∗∗^	.025
DPPHi					1	-.148	.493^∗∗^	.729^∗∗^	.003	.670^∗∗^	.269^∗∗^	.459^∗∗^	.106
FRAPi						1	-.237^∗^	-.068	.279^∗∗^	-.111	-.257^∗∗^	-.350^∗∗^	.016
PPs							1	.378^∗∗^	-.042	.889^∗∗^	.784^∗∗^	.664^∗∗^	.147
FLs								1	.296^∗∗^	.720^∗∗^	.534^∗∗^	.620^∗∗^	.584^∗^
TTs									1	.408^∗∗^	.527^∗∗^	.382^∗∗^	.339^∗∗^
ABTSs										1	.641^∗∗^	.690^∗∗^	.464^∗∗^
DPPHs											1	.908^∗∗^	.383^∗∗^
FRAPs												1	.279^∗∗^
AT													1

^∗∗^Correlation is significant at the 0.01 level. ^∗^Correlation is significant at the 0.05 level. PPi: bound polyphenols; FLi: bound flavonoids; TTi: bound tannins; ABTSi: ABTS activity of the bound phenolic compounds; DPPHi: DPPH activity of the bound phenolic compounds; FRAPi: FRAP activity of the bound phenolic compounds; PPs: free polyphenols; FLs: free flavonoids; TTs: free tannins; ABTSs: ABTS activity of the free phenolic compounds; DPPHs: DPPH activity of the free phenolic compounds; FRAPs: FRAP of the free phenolic compounds; TA: total anthocyanins.

## Data Availability

The data that support the findings of this study are available from the corresponding author, [LA], upon reasonable request.
